# Editorial Comment: Two-stage Fowler-Stephens orchidopexy in management of undescended testes: Is it time for a change? A UK multi-centre retrospective study

**DOI:** 10.1590/S1677-5538.IBJU.2025.9923

**Published:** 2025-12-26

**Authors:** Luciano A. Favorito

**Affiliations:** 1 Universidade do Estado do Rio de Janeiro Unidade de Pesquisa Urogenital Rio de Janeiro RJ Brasil Unidade de Pesquisa Urogenital - Universidade do Estado do Rio de Janeiro - Uerj, Rio de Janeiro, RJ, Brasil; 2 Hospital Federal da Lagoa Serviço de Urologia Rio de Janeiro RJ Brasil Serviço de Urologia, Hospital Federal da Lagoa, Rio de Janeiro, RJ, Brasil

**Hirokazu Ikeda ^1^, Yoshitaka Watanabe ^1^, Yoshiyuki Ohtomo ^2^, Hiroki Miyano ^2^, Shuichiro Fujinaga ^3^, Yusuke Gonda ^3^, et al.**

^1^ Children's Medical Center, Showa Medical University Northern Yokohama Hospital, Kanagawa, Japan;

^2^ Department of Pediatrics, Juntendo University Nerima Hospital, Tokyo, Japan; ^3^ Divisions of Nephrology, Saitama Children's Medical Center, Saitama, Japan.

**Transl Pediatr. 2025 Nov 30;14(11):2955-2967.**

DOI: 10.21037/tp-2025-573 | ACCESS: 41367527

## COMMENT

Ikeda et al. (1) present an interesting perspective on the use of β3-adrenoceptor agonists in the treatment of nocturnal enuresis. These agents have emerged as a promising therapeutic option in the management of nocturnal enuresis (NE), particularly in patients with bladder overactivity (2, 3). β3-adrenoceptor agonists act by selectively stimulating β3-adrenergic receptors located in the detrusor muscle of the urinary bladder. Activation of these receptors promotes detrusor relaxation during the storage phase, thereby increasing functional bladder capacity. As a result, involuntary bladder contractions occurring during sleep may be reduced (4, 5).

In nocturnal enuresis—especially when associated with nocturnal polyuria or reduced bladder capacity—β3-adrenoceptor agonists may help stabilize bladder function overnight (3, 4). Unlike anticholinergic agents, they do not inhibit muscarinic receptors, which reduces the risk of common adverse effects such as dry mouth, constipation, and cognitive impairment. This favorable safety profile is particularly relevant in pediatric and adolescent populations.

In children and adolescents with enuresis, especially those with underlying bladder overactivity or reduced bladder capacity, mirabegron may help reduce involuntary detrusor contractions during sleep. By improving bladder storage and reducing nocturnal urgency, the drug may decrease the frequency of bedwetting episodes. Its use has been reported mainly as an off-label treatment, often in patients refractory to standard therapies such as desmopressin or anticholinergic agents (3, 4).

The present study evaluated 387 children aged 5–18 years who received vibegron (50 mg once daily) for refractory nocturnal enuresis. The authors concluded that vibegron is a safe and effective option for pediatric patients with treatment-resistant NE. Add-on strategies—particularly triple therapy—were more effective than switching monotherapy, supporting the incorporation of vibegron as part of multimodal treatment approaches. Given the retrospective design of the study, prospective randomized trials are warranted to confirm these findings and to optimize treatment protocols.

## Data Availability

All data generated or analysed during this study are

## References

[1] Ikeda H, Watanabe Y, Othomo Y, Sato K, Tanaka M, Fujimoto T (2025). Safety and efficacy of vibegron in pediatric patients with treatment-resistant nocturnal enuresis: a multicenter retrospective study. Transl Pediatr.

[2] Dutra MF, Lima EM, Murillo JB, de Paula LIDS, de Bessa J, Pereira ALA (2025). Parasacral Transcutaneous Electrical Nerve Stimulation with Desmopressin Acetate for Treating Primary Monosymptomatic Enuresis: A Randomized Controlled Clinical Trial. Int Braz J Urol.

[3] Mansour I, Laymon M, Abdelhalim A, Dawaba MS, El-Hefnawy AS (2025). Efficacy and Safety of Mirabegron Compared to Solifenacin in Treatment of Non-neurogenic Overactive Bladder in Children: A Randomized Controlled Trial. Int Braz J Urol.

[4] Dutra MF, Bessa J, Almeida ECL, Lima EM, Netto JBM, Silva RF (2024). The effectiveness of parasacral transcutaneous electrical nerve stimulation in the treatment of monosymptomatic enuresis in children and adolescents: a systematic review. Int Braz J Urol.

[5] Braga AVNM, Nunes NC, Santos EN, Heldwein FL, Srougi M, Fornari A (2024). Use of ChatGPT in urology and its relevance in clinical practice: is it useful?. Int Braz J Urol.

